# Genome-wide analysis of the WRKY gene family and their positive responses to phytoplasma invasion in Chinese jujube

**DOI:** 10.1186/s12864-019-5789-8

**Published:** 2019-06-07

**Authors:** Chaoling Xue, Hongtai Li, Zhiguo Liu, Lili Wang, Yitong Zhao, Ximeng Wei, Hu Fang, Mengjun Liu, Jin Zhao

**Affiliations:** 1 College of Life Science, Hebei Agricultural University, Baoding, China; 2 Key Laboratory of Hebei Province for Plant Physiology and Molecular Pathology, Hebei Agricultural University, Baoding, Hebei China; 3 Research Center of Chinese Jujube, Hebei Agricultural University, Baoding, China; 4 BGI-Shenzhen, Shenzhen, China

**Keywords:** WRKYs, Chinese jujube, Bioinformatics analysis, Gene expression, Phytoplasma invasion

## Abstract

**Background:**

The WRKY gene family is one of the most important families in higher plants. As transcription factors, they actively respond to biotic and abiotic stress and are also involved in plant development. Chinese jujube (*Ziziphus jujuba* Mill.) is the largest type of dried fruit tree in China in terms of production, but its production is largely limited by phytoplasma infection, and the information about the role of *WRKY* genes under phytoplasma stress was still limited.

**Results:**

We identified 54 *ZjWRKYs* in the jujube genome and classified them into three subgroups according to conserved WRKY domains and zinc-finger structure. 41 *ZjWRKYs* were distributed on 11 of 12 pseudo chromosomes in Chinese jujube. The majority of *ZjWRKYs* were highly expressed in the seven examined tissues, indicating that they play multiple roles in these vegetative and reproductive organs. Transcriptome data showed that most of the characterised *ZjWRKYs* were highly expressed at later stages of fruit development. RT-qPCR demonstrated that the expression of 23 *ZjWRKYs* changed following phytoplasma infection, suggesting that they are involved in signalling pathways that respond to phytoplasma stress. Then, STRING analysis and yeast two-hybrid screening proved that some ZjWRKY proteins were interacting with ZjMAPKK proteins, which were also involved in phytoplasma invasion. Moreover, their differential expressions were further confirmed in resistant and susceptible jujube varieties under phytoplasma stress. These results suggest that *ZjWRKYs* play significant roles in phytoplasma tolerance and should be crucial candidate genes for jujube-phytoplasma interaction.

**Conclusions:**

54 *ZjWRKYs* in Chinese jujube were identified and classified into three subgroups. 41 *ZjWRKYs* were unevenly distributed along the chromosomes. The majority of *ZjWRKYs* were highly expressed in various tissues. Most of the *ZjWRKYs* were positive responses to phytoplasma invasion, and that provided candidate genes for the future studies of jujube-phytoplasma interaction.

**Electronic supplementary material:**

The online version of this article (10.1186/s12864-019-5789-8) contains supplementary material, which is available to authorized users.

## Background

The WRKY transcription factors (TFs) bind to a specific promoter sequence in the target gene, known as a W-box, and can positively or negatively regulate target gene expression. The WRKY proteins have one or two DNA binding domains that are 60 amino acids long and contain the conserved heptapeptide WRKYGQK followed by a zinc-finger motif C_2_H_2_ (CX_4-5_CX_22–23_HXH) or C_2_HC (CX_7_CX_23–24_ HXC) [1]. The WRKY family contains important transcription factors that have multiple functions in processes such as embryogenesis [2], trichome and seed development [3], leaf senescence [4], flowering [5], fruit and pollen development [6], biomass accumulation [7], secondary metabolite biosynthesis [8] and hormone signalling [9]. WRKY transcription factors are also crucial regulatory components of plant responses to pathogen infection. In Arabidopsis, several *WRKY* genes have been experimentally characterised and associated with responses to fungal or bacterial pathogens [10–12]. AtWRKY70 is required for R gene-mediated pathogen resistance, determining the balance between the SA- and JA-dependent defence systems [13, 14]. Many *WRKY* genes also act in defence signalling; for example, *AtWRKY38* and *AtWRKY62* act as negative regulators of basal resistance towards bacterial pathogens [15]. In rice, overexpression of *OsWRKY30* enhanced resistance to the rice sheath blight fungus *Rhizoctonia solani* and the blast fungus *Magnaporthe grisea* [16, 17]. Owing to their important roles, the WRKY family has been widely studied in many plant species, such as Arabidopsis, rice, grape, apple, pear, and peach [18–22]. However, the information of this gene family in Chinese jujube and their roles under phytoplasma stress was still limited.

Chinese jujube is the largest type of dried fruit tree in China in terms of production [23] and the most important species of family Rhamnaceae. It is cultivated mainly for its fruits, which can be eaten fresh or dried or as raw materials for making Chinese herbal medicine. However, jujube production is threatened by several devastating diseases, such as jujube witches’ broom disease (JWB). The genome of Chinese jujube was recently published [24, 25], paving the way for further investigations. Our transcriptome data indicated that some *WRKY* genes respond to JWB phytoplasma infection. Since the WRKY family plays a crucial role in biotic stress response, identifying *WRKY* genes in Chinese jujube and determining their possible functions in response to phytoplasma stress have important significance.

Here, we report on the genome-wide analysis of the WRKY family in Chinese jujube. A non-redundant set of *WRKY* genes was identified in this species. Subsequently, chromosomal location was determined, phylogenetic and motif analyses were also performed as a base for further comparative genomics studies. Moreover, expression patterns of *ZjWRKY*s in various tissues and under phytoplasma stress were also investigated. The interacting proteins of ZjWRKYs were also screened. The ZjWRKYs involved in phytoplasma invasion were considered good candidates for subsequent studies of the jujube-phytoplasma interaction.

## Results

### Identification of ZjWRKYs in Chinese jujube

A total of 54 non-redundant putative WRKY coding sequences (Table 1)

**Table 1:** The information of WRKY gene family in Chinese jujube

Gene Name	NCBI Reference	ORF (bp)	Size (aa)	MW(D)	PI	Conserved motif	Domain pattern	Zinc finger	Group	Exon number
*ZjWRKY1*	XM_016044069.1	1434	477	52053.75	9.09	2 × [WRKYGQK]	C-X_4_-C-X_22–23_-HXH	C_2_H_2_	I	5
*ZjWRKY2*	XM_016042179.1	1629	542	60186.57	7.09	2 × [WRKYGQK]	C-X_4_-C-X_22–23_-HXH	C_2_H_2_	I	5
*ZjWRKY3*	XM_016047139.1	1904	583	63795.60	6	2 × [WRKYGQK]	C-X_4_-C-X_22–23_-HXH	C_2_H_2_	I	6
*ZjWRKY4*	XM_016025559.1	1506	501	55188.92	6.52	2 × [WRKYGQK]	C-X_4_-C-X_22–23_-HXH	C_2_H_2_	I	4
*ZjWRKY5*	XM_016037165.1	1566	521	57263.14	5.12	2 × [WRKYGQK]	C-X_4_-C-X_22–23_-HXH	C_2_H_2_	I	5
*ZjWRKY6*	XM_016019228.1	1077	358	39860.32	8.82	2 × [WRKYGQK]	C-X_4_-C-X_22–23_-HXH	C_2_H_2_	I	4
*ZjWRKY7*	XM_016024358.1	1629	542	59088.72	8.91	2 × [WRKYGQK]	C-X_4_-C-X_22–23_-HXH	C_2_H_2_	I	5
*ZjWRKY8*	XM_016020284.1	2205	734	80415.39	5.9	2 × [WRKYGQK]	C-X_4_-C-X_22–23_-HXH	C_2_H_2_	I	1
*ZjWRKY9*	XM_016029235.1	951	316	34758.07	8.44	WRKYGQK	C-X_5_-C-X_23_-HXH	C_2_H_2_	IIa	4
*ZjWRKY10*	XM_016014490.1	951	316	35076.43	8.68	WRKYGQK	C-X_5_-C-X_23_-HXH	C_2_H_2_	IIa	5
*ZjWRKY11*	XM_016028547.1	801	266	29844.33	8.99	WRKYGQK	C-X_5_-C-X_23_-HXH	C_2_H_2_	IIa	4
*ZjWRKY12*	XM_016022282.1	1863	620	67027.91	6.26	WRKYGQK	C-X_5_-C-X_23_-HXH	C_2_H_2_	IIb	5
*ZjWRKY13*	XM_016029844.1	1902	633	67897.98	6.12	WRKYGQK	C-X_5_-C-X_23_-HXH	C_2_H_2_	IIb	6
*ZjWRKY14*	XM_016043879.1	1611	536	58692.78	6.48	WRKYGQK	C-X_5_-C-X_23_-HXH	C_2_H_2_	IIb	6
*ZjWRKY15*	XM_016036346.1	1125	374	40424.14	8.09	WRKYGQK	C-X_5_-C-X_23_-HXH	C_2_H_2_	IIb	3
*ZjWRKY16*	XM_016036345.1	1659	552	60686	7.71	WRKYGQK	C-X_5_-C-X_23_-HXH	C_2_H_2_	IIb	7
*ZjWRKY17*	XM_016037515.1	1512	503	56033.17	5.48	WRKYGQK	C-X_5_-C-X_23_-HXH	C_2_H_2_	IIb	5
*ZjWRKY18*	XM_016039435.1	1251	416	44528.06	9.13	WRKYGQK	C-X_5_-C-X_23_-HXH	C_2_H_2_	IIb	3
*ZjWRKY19*	XM_016014870.1	1878	625	67519.49	7.97	WRKYGQK	C-X_5_-C-X_23_-HXH	C_2_H_2_	IIb	5
*ZjWRKY20*	XM_016014977.1	1764	587	63426.84	8.8	WRKYGQK	C-X_5_-C-X_23_-HXH	C_2_H_2_	IIb	4
*ZjWRKY21*	XM_016014513.1	1878	625	67533.52	7.97	WRKYGQK	C-X_5_-C-X_23_-HXH	C_2_H_2_	IIb	5
*ZjWRKY22*	XM_016038792.1	579	192	21894.40	9.43	WRKYGQK	C-X_4_-C-X_23_-HXH	C_2_H_2_	IIc	2
*ZjWRKY23*	XM_016040974.1	636	211	24030.21	8.47	WRKYGQK	C-X_4_-C-X_23_-HXH	C_2_H_2_	IIc	3
*ZjWRKY24*	XM_016026686.1	597	198	22899.66	9.23	WRKYGQK	C-X_4_-C-X_23_-HXH	C_2_H_2_	IIc	2
*ZjWRKY25*	XM_016014637.1	588	195	21527.72	6.73	WRKYGKK	C-X_4_-C-X_23_-HXH	C_2_H_2_	IIc	3
*ZjWRKY26*	XM_016028953.1	522	173	19750.96	5.59	WRKYGKK	C-X_4_-C-X_23_-HXH	C_2_H_2_	IIc	3
*ZjWRKY27*	XM_016011550.1	1008	335	37240.76	6.34	WRKYGQK	C-X_4_-C-X_23_-HXH	C_2_H_2_	IIc	4
*ZjWRKY28*	XM_016024581.1	1038	345	39158.92	6.76	WRKYGQK	C-X_4_-C-X_23_-HXH	C_2_H_2_	IIc	3
*ZjWRKY29*	XM_016039492.1	933	310	34331.69	5.65	WRKYGQK	C-X_4_-C-X_23_-HXH	C_2_H_2_	IIc	3
*ZjWRKY30*	XM_016011683.1	1110	369	40614.00	5.16	WRKYGQK	C-X_4_-C-X_23_-HXH	C_2_H_2_	IIc	3
*ZjWRKY31*	XM_016036211.1	1008	335	37135.37	6.43	WRKYGQK	C-X_4_-C-X_23_-HXH	C_2_H_2_	IIc	3
*ZjWRKY32*	XM_016041473.1	756	251	28026.78	4.65	WRKYGQK	C-X_4_-C-X_23_-HXH	C_2_H_2_	IIc	2
*ZjWRKY33*	XM_016028867.1	1116	371	40189.57	9.57	WRKYGQK	C-X_5_-C-X_23_-HXH	C_2_H_2_	IId	3
*ZjWRKY34*	XM_016011768.1	1101	366	39383.33	9.64	WRKYGQK	C-X_5_-C-X_23_-HXH	C_2_H_2_	IId	3
*ZjWRKY35*	XM_016036017.1	1083	360	40498.69	9.65	WRKYGQK	C-X_5_-C-X_23_-HXH	C_2_H_2_	IId	4
*ZjWRKY36*	XM_016045200.1	1497	498	53218.21	5.81	WRKYGQK	C-X_5_-C-X_23_-HXH	C_2_H_2_	IIe	3
*ZjWRKY37*	XM_016020139.1	858	285	31393.44	5.62	WRKYGQK	C-X_5_-C-X_23_-HXH	C_2_H_2_	IIe	3
*ZjWRKY38*	XM_016025812.1	849	282	30475.83	5.46	WRKYGQK	C-X_5_-C-X_23_-HXH	C_2_H_2_	IIe	3
*ZjWRKY39*	XM_016022820.1	1422	473	51502.28	5.19	WRKYGQK	C-X_5_-C-X_23_-HXH	C_2_H_2_	IIe	3
*ZjWRKY40*	XM_016044078.1	969	322	36328.33	8.98	WRKYGQK	C-X_5_-C-X_23_-HXH	C_2_H_2_	IIe	4
*ZjWRKY41*	XM_016044080.1	1068	355	38920.61	5.92	WRKYGQK	C-X_5_-C-X_23_-HXH	C_2_H_2_	IIe	3
*ZjWRKY42*	XM_016036213.1	870	289	32200.49	5.27	WRKYGQK	C-X_5_-C-X_23_-HXH	C_2_H_2_	IIe	3
*ZjWRKY43*	XM_016013400.1	1215	404	45202.30	6.64	WRKYGQK	C-X_7_-C-X_23_-HXC	C_2_HC	III	4
*ZjWRKY44*	XM_016040779.1	1113	370	41825.52	5.26	WRKYGQK	C-X_7_-C-X_23_-HXC	C_2_HC	III	3
*ZjWRKY45*	XM_016022705.1	1176	391	43607.65	5.9	WRKYGQK	C-X_7_-C-X_23_-HXC	C_2_HC	III	3
*ZjWRKY46*	XM_016033893.1	1065	354	40462.32	5.16	WRKYGQK	C-X_7_-C-X_23_-HXC	C_2_HC	III	4
*ZjWRKY47*	XM_016041850.1	960	319	36005.79	5.25	WRKYGQK	C-X_7_-C-X_23_-HXC	C_2_HC	III	3
*ZjWRKY48*	XM_016013401.1	1035	344	38711.93	5.39	WRKYGQK	C-X_7_-C-X_23_-HXC	C_2_HC	III	3
*ZjWRKY49*	XM_016041861.1	981	326	37477.17	8.15	WRKYGQK	C-X_7_-C-X_23_-HXC	C_2_HC	III	3
*ZjWRKY50*	XM_016041806.1	930	309	35609.87	5.91	WRKYGQK	C-X_7_-C-X_23_-HXC	C_2_HC	III	3
*ZjWRKY51*	XM_016047371.1	924	307	35268.77	6.71	WRKYGQK	C-X_7_-C-X_23_-HXC	C_2_HC	III	3
*ZjWRKY52*	XM_016041801.1	948	315	35731.33	6.46	WRKYGQK	C-X_7_-C-X_23_-HXC	C_2_HC	III	3
*ZjWRKY53*	XM_016041802.1	972	323	37104.55	8.78	WRKYGQK	C-X_7_-C-X_23_-HXC	C_2_HC	III	3
*ZjWRKY54*	XM_016041803.1	951	316	36421.82	5.76	WRKYGQK	C-X_7_-C-X_23_-HXC	C_2_HC	III	3

were identified in the jujube genome sequence. The sequences were named from *ZjWRKY1* to *ZjWRKY54* according to their gene structure and motifs. The ORF length for *ZjWRKY* genes ranged from 522 bp (*ZjWRKY26*) to 2205 bp (*ZjWRKY8*), and they encoded proteins ranging from 173 to 734 amino acids (aa) in length, with predicted pIs ranging from 4.65 (*ZjWRKY32*) to 9.09 (*ZjWRKY1*) (Table 1).

Previous genome evolution studies showed that Chinese jujube is closely related to species of the family Rosaceae [24, 26], so the *WRKY* genes of three Rosaceae species (apple, pear and peach) and Arabidopsis were compared with that of Chinese jujube (Additional file 1). Compared with Arabidopsis, apple and pear [18, 19, 21], there are fewer WRKY genes in jujube, but the number was similar to that of peach [22]. The smaller number of *WRKY* genes in Chinese jujube and peach may be due to the occurrence of only one genome duplication event during the evolution of the two species [24, 27]. Based on the above comparison, it was suggested that most of the expected *WRKY* genes in jujube were identified.

### Conserved motifs and phylogenetic tree construction of ZjWRKYs

**Fig. 1:**
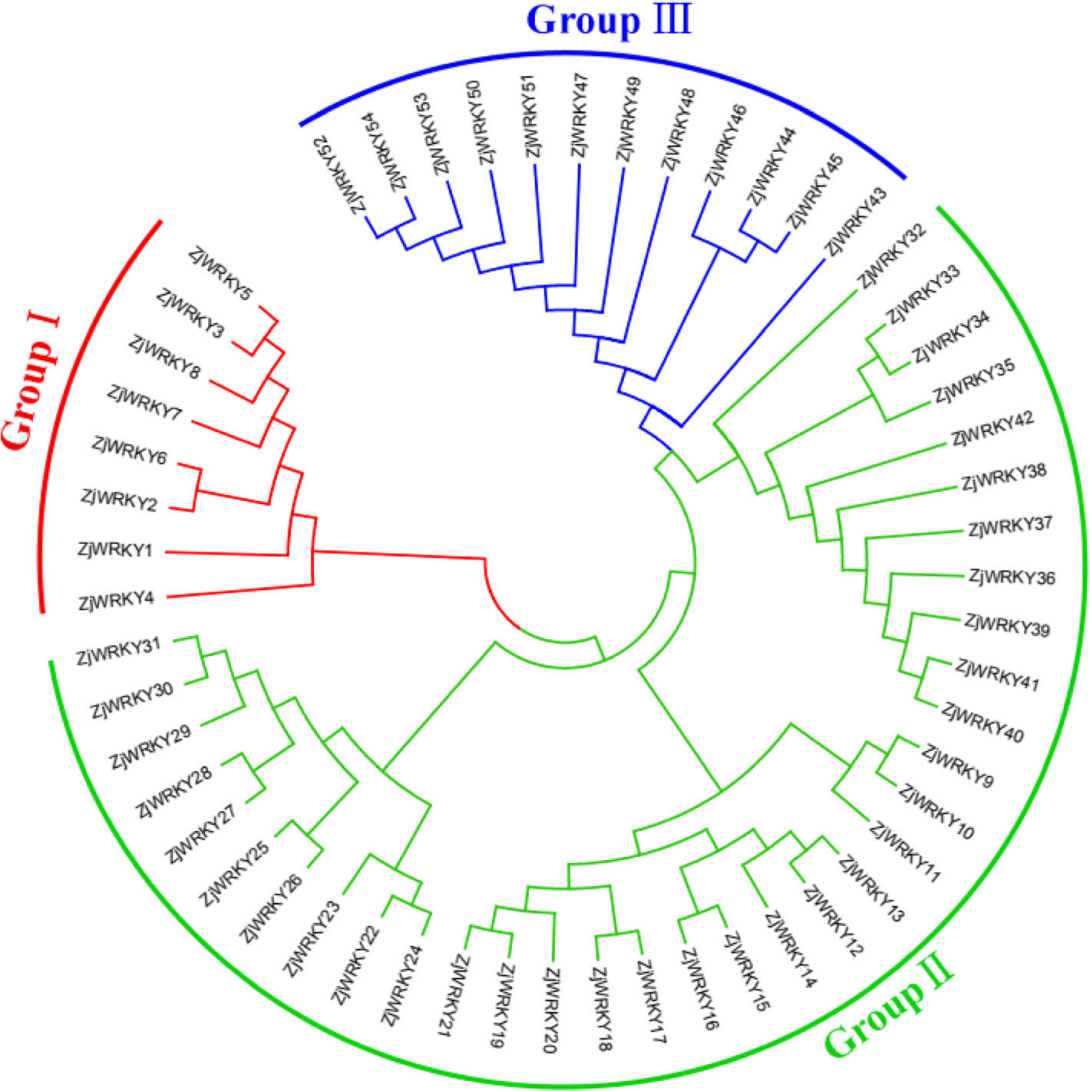
The phylogenetic tree of the ZjWRKY proteins. The NJ tree was constructed from the amino acid sequences of ZjWRKYs using MEGA5.2 with 1000 bootstrap replicates

**Fig. 2:**
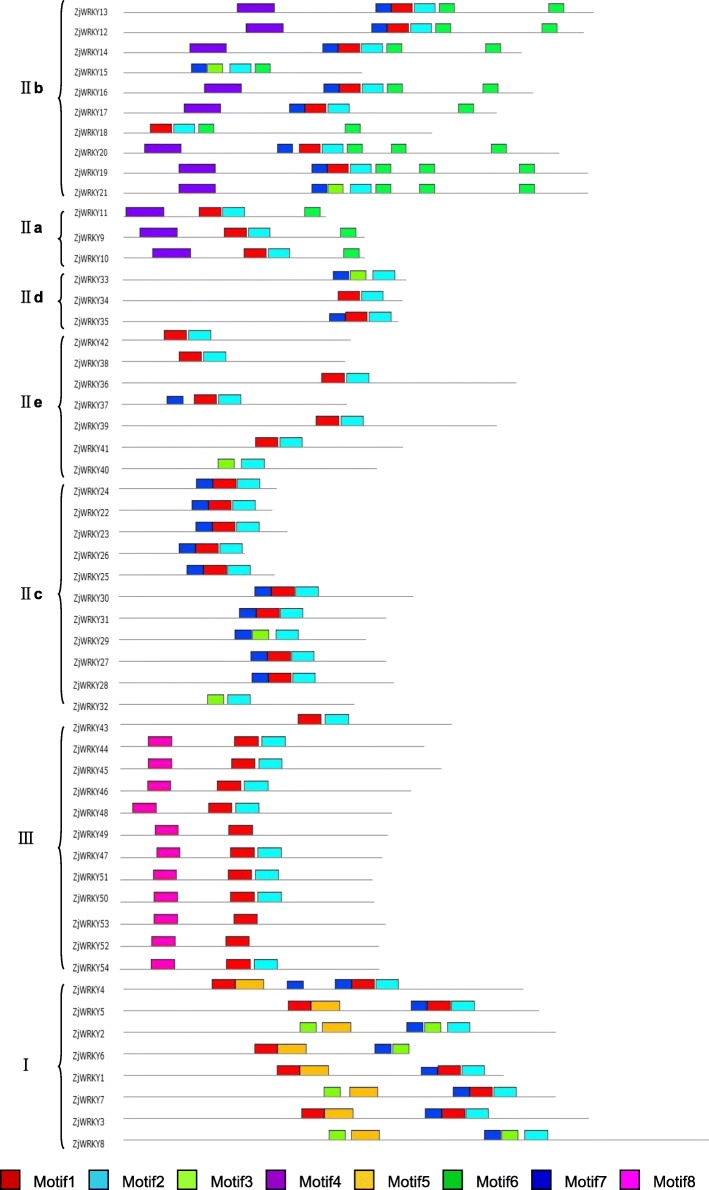
Conserved motifs of the ZjWRKY proteins arranged according to their phylogenetic relationships. The motifs in the ZjWRKYs were identified using Multiple Em for Motif Elicitation (MEME). In ZjWRKY proteins, 8 conserved motifs were identified and shown in different colors

The phylogenetic tree of the ZjWRKY proteins was constructed by aligning multiple domain sequences (Fig. 1). The ZjWRKY proteins were classified into three groups (Group I, II and III) (Table 1) according to their WRKY and zinc-finger motifs. The domain sequences in the ZjWRKY gene family were highly conserved. There were 8 motifs among ZjWRKYs and proteins in the same group had similar numbers and types of motifs (Fig. 2, Additional file 2). The WRKY domain (WRKYGQK, Motif 1) was highly conserved among the 54 proteins (Additional file 2) and only two of them contained variations. The group II proteins ZjWRKY25 and ZjWRKY26 showed a WRKY motif with one amino acid modifications (WRKYGKK) (Table 1, Fig. 2). Motif 2 was also highly conserved except in the two Group III proteins ZjWRKY52 and ZjWRKY53. Motif 5 and Motif 8 were specific to groups I and III respectively.

Group I had 8 proteins (Table 1), that contained two WRKY motifs, and two C_2_H_2_ zinc-finger motifs. Group II was the biggest group and included 34 proteins that contained a WRKY motif and a C_2_H_2_ zinc-finger motif. According to the phylogenetic analysis, the 34 genes could be further divided into five subgroups (IIa to e) that included 3, 10, 11, 3 and 7 genes, respectively (Table 1). The members of subgroups IIa, IIb, IId and IIe had a CX_5_CX_23_HX_1_H zinc-finger motif, while that of subgroup IIc had a CX_4_CX_23_HX_1_H structure (Table 1). Group III contained 12 proteins, and they had one WRKY motif and a C_2_HC zinc-finger motif (CX_7_CX_23_HX_1_C, Table 1).

### The chromosomal location and gene structure of ZjWRKYs

**Fig. 3:**
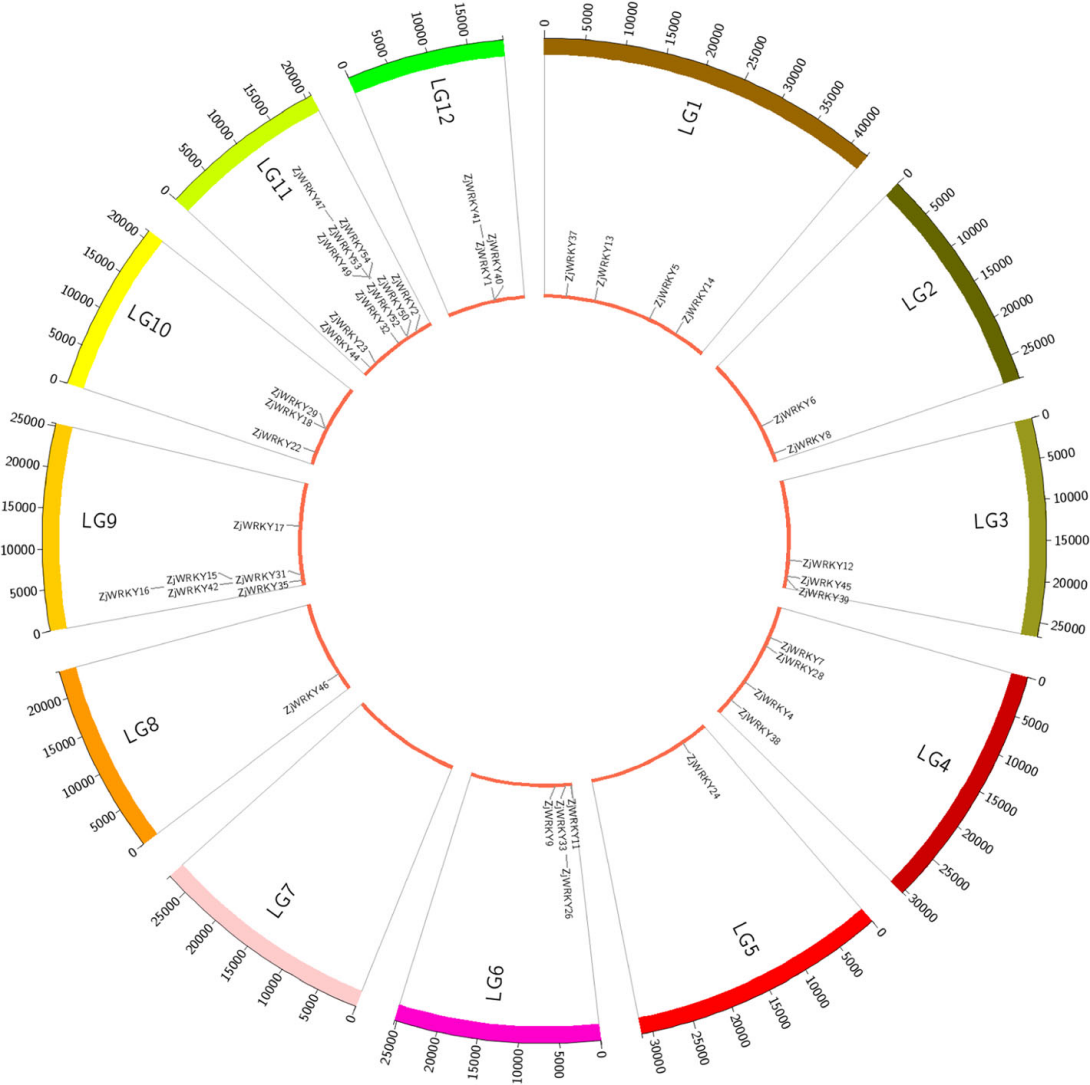
Positions of 41 *ZjWRKY* genes on the jujube chromosomes. Genes were mapped to the jujube chromosomes via the Circos tool. The jujube chromosomes were arranged in a circle

**Fig. 4:**
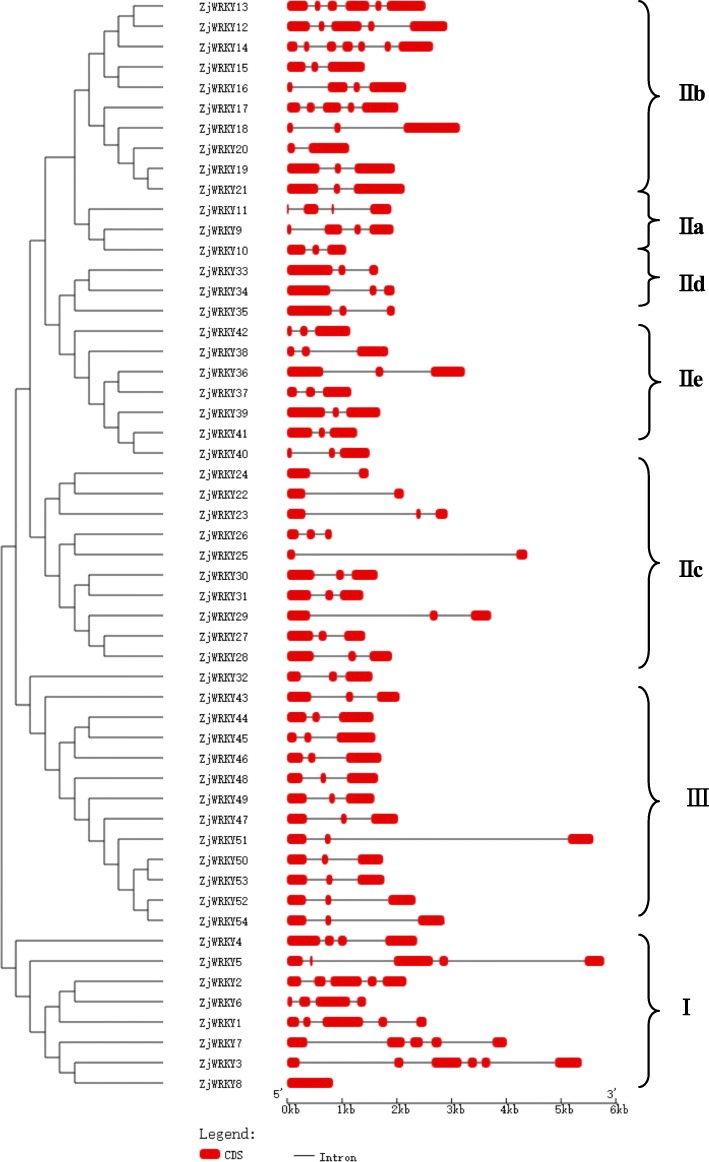
The exon/intron structure of 54 Z*jWRKY* genes in Chinese jujube. Introns and exons are represented by black lines and red boxes respectively

Of the 54 *ZjWRKY* genes, 41 were mapped to 11 of 12 pseudo chromosomes in the jujube genome (Fig. 3), and 13 genes were located on 12 scaffolds (Table 1, Additional file 3). *ZjWRKYs* were not evenly distributed across the 11 pseudo chromosomes (Fig. 3). Ten *ZjWRKYs* (18.5%) were located on Chr. 11, whereas only one *ZjWRKY* gene was on Chr. 5 and 8 each. No *ZjWRKY* gene was found on Chr. 7. Additionally, the gene structure was highly conserved within each group, especially in groups IId, IIe, and III. We found that Group I genes contained more introns and were more complicated than genes in the other two groups (Fig. 4). Tandem duplications were present in 40.7% of *ZjWRKY* genes (*ZjWRKY1*, *15*, *16*, *18, 26*, *29*, *31*, *33, 35*, *39*, *40*, *41*, *42, 45, 47*, *49, 50*, *52*, *53*, and *54*), which contributed to the expansion of the *ZjWRKY* gene family. This dynamic was particularly evident in Group III in which 8 out of the 12 genes (66.7%) mapped to duplicated chromosome or scaffold regions.

### Expression profiles of *ZjWRKYs* in various tissues/organs

**Fig. 5:**
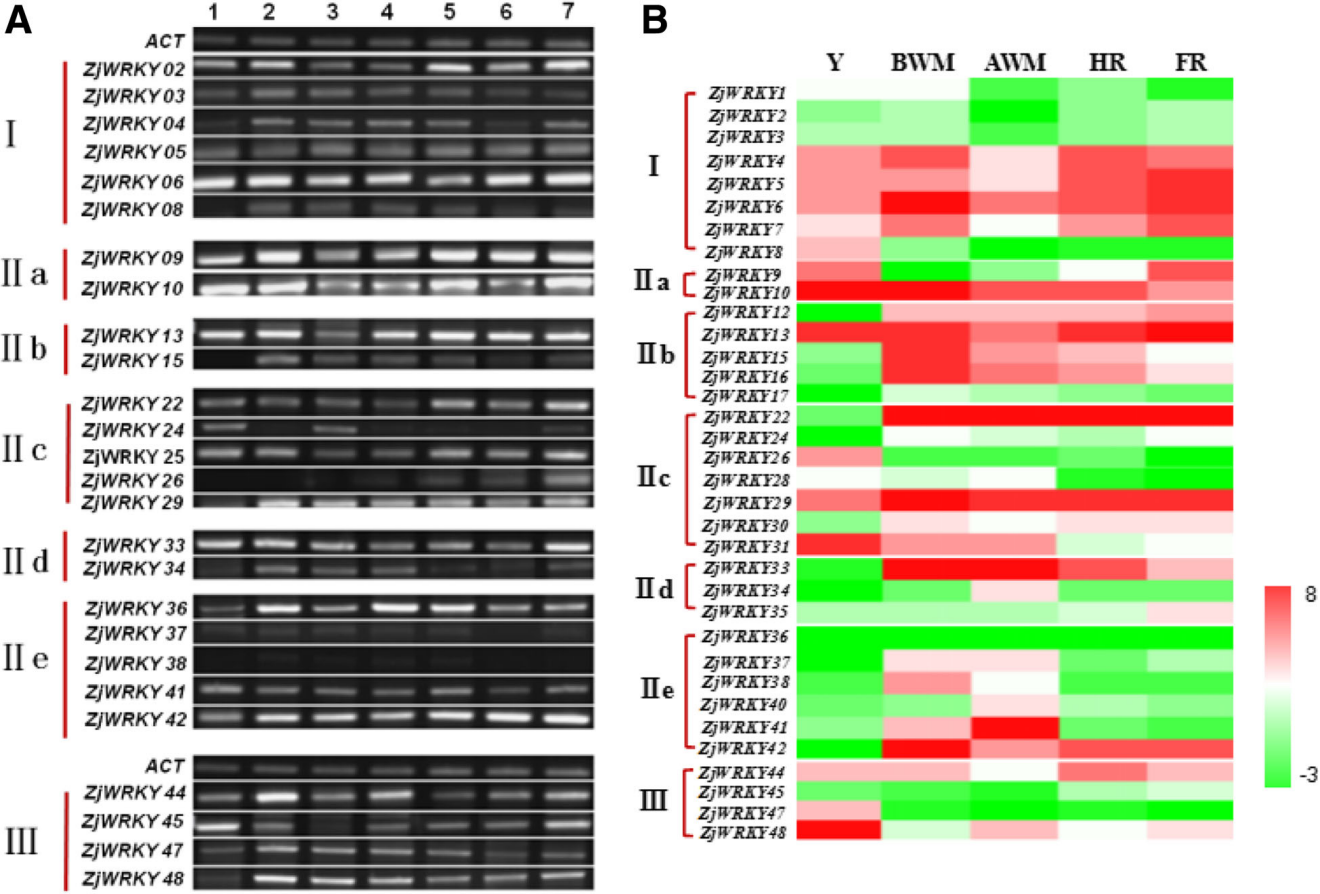
**a** Expression pattern of *ZjWRKY* genes in seven tissues/organs by RT-PCR. *ZjACT* was used as an internal control. From left to right: root, bearing shoot, secondary shoot, leaf, flower bud, flower, and fruit. **b** Heat map of RNA-Seq data for *WRKY* genes during jujube fruit ripening. Y, young fruit; BWM, before white mature fruit; WM, white mature fruit; HR, half-red fruit; FR, full red fruit. Scaled log2 expression values are shown from green to red, indicating low to high expression

To investigate the tissue-specific expression of the jujube *WRKY* genes, RT-PCR was used to determine their expression patterns in seven tissues. The expression patterns of 26 *ZjWRKY* genes were analysed and are shown in Fig. 5a. Of the 26 *ZjWRKY* genes, six genes were actively expressed in at least five tissues, including *ZjWRKY6*, *9*, *10*, *13*, *29,* and *42*. A total of 8 *ZjWRKY* genes (*ZjWRKY2*, *22*, *25*, *33*, *36*, *44*, *45* and *48*) were found to be upregulated in only one or two tissues, indicative of the tissue-specific expression of these genes. *ZjWRKY24* expression could only be detected in roots and old branches. The expression of the remaining genes was comparatively low in the different organs, suggesting that genes in the same group might have different functions. These results showed that most of the *ZjWRKY* genes had diverse tissue-specific expression patterns, indicating that *ZjWRKYs* play multiple roles in various organs.

Moreover, a heat map of our RNA-Seq data highlighted differential expression of *ZjWRKYs* during jujube fruit development (Fig. 5b), and most of the genes were expressed at different levels. The genes of group IIe were mainly expressed at before white mature period (BWM) and white mature period (WM), and the expression of group IIb genes was lower at young fruit period (Y) except for *ZjWRKY13*. *ZjWRKY8*, *26*, *47*, and *48* were only involved in the development of young fruit, suggesting the role for these *WRKY* genes in jujube fruit development.

### *ZjWRKYs* involved in the jujube-phytoplasma interaction

**Fig. 6:**
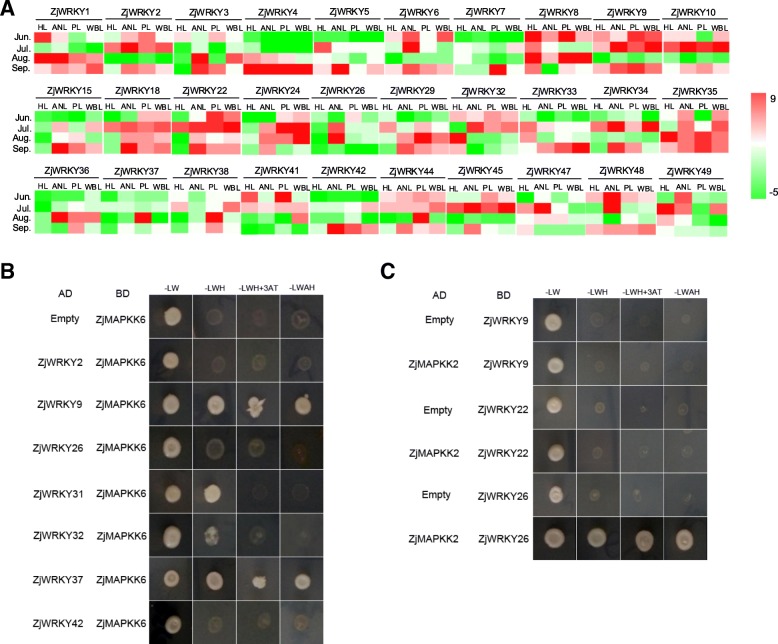
**a** Heat map of relative expression of *WRKY* genes under phytoplasma stress. Scaled log2 expression values are shown from green to red, indicating low to high expression. (**b** and **c**) Yeast two-hybrid screening of ZjWRKYs and ZjMAPKK2/ZjMAPKK6

The expression of the phytoplasma *TMK* gene was not detected in the healthy leaves, however its highly expression was found in other diseased tissues, including the apparently normal leaves (Additional file 4). Among the 30 *ZjWRKY* genes detected, 17 *ZjWRKYs* (*ZjWRKY2*, *3*, *6*, *9*, *10*, *15*, *18*, *22*, *24*, *26*, *34*, *36*, *37*, *38*, *42*, *44*, and *45*) were significantly upregulated under phytoplasma stress, while 5 *ZjWRKYs* (*ZjWRKY5*, *8*, *33*, *47*, and *49*) were downregulated (Fig. 6a). The expression of *ZjWRKY32* first increased and then decreased in diseased jujube. Most Group II genes were upregulated under phytoplasma stress. *ZjWRKY37*, *ZjWRKY38,* and *ZjWRKY44* were significantly upregulated in phyllody leaves. *ZjWRKY5* and *ZjWRKY49* were significantly downregulated in diseased leaves. These *ZjWRKY* genes displayed noticeable changes in expression and should play vital roles in jujube-phytoplasma interactions.

STRING analysis displayed that WRKY proteins could function by interacting with each other, as well as with MPK3 (Additional file 7A). Furtherly, yeast two-hybrid screening proved that ZjWRKY9 and ZjWRKY37 were interacting with ZjMAPKK6 (Fig. 6b), and ZjWRKY26 was interacting with ZjMAPKK2 (Fig. 6c). In previous study, it was found that ZjMAPKs and ZjMAPKKs were also involved in phytoplasma infection [28].

**Fig. 7:**
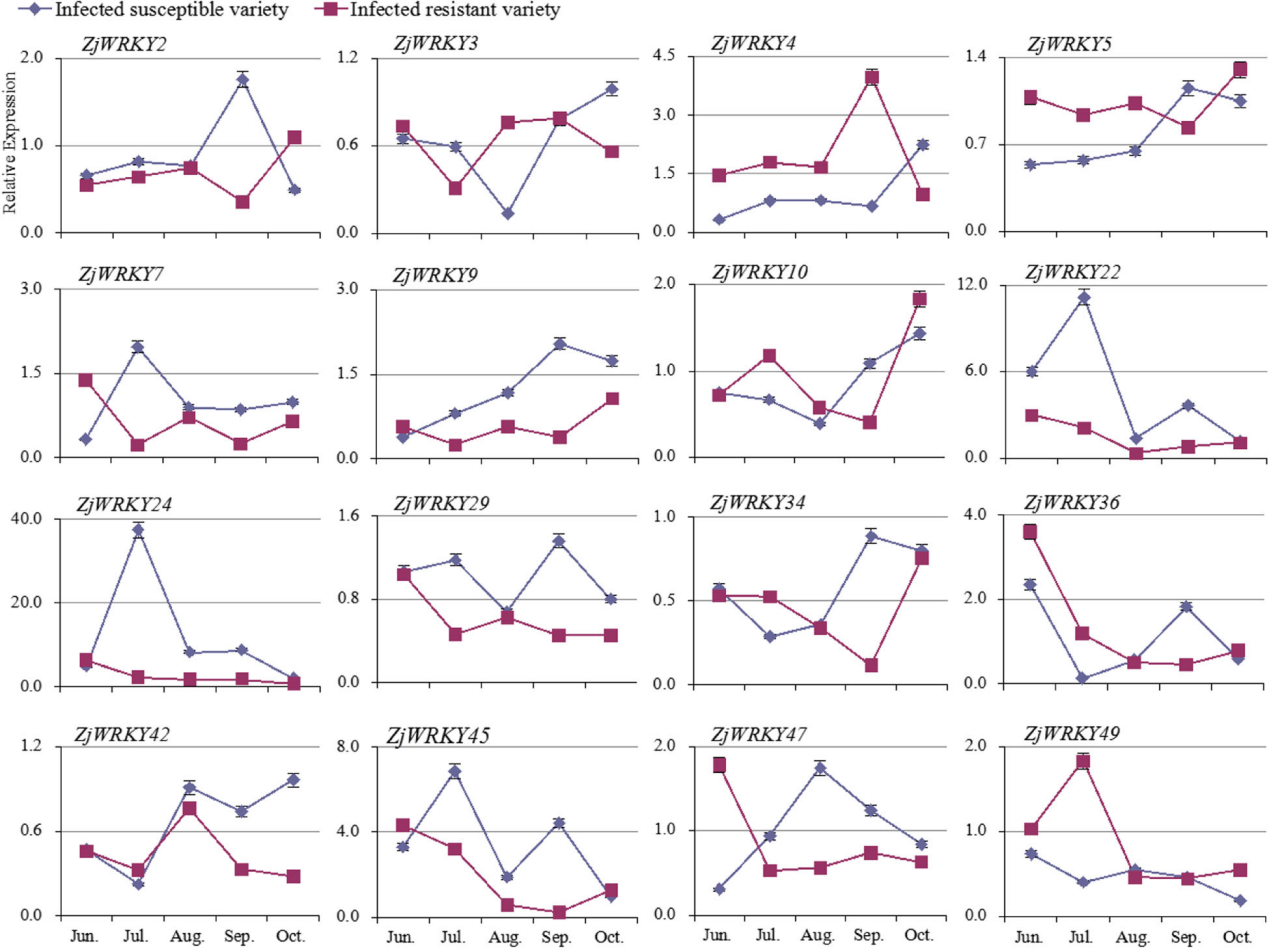
Relative expression of *ZjWRKY* genes in JWB-resistant and susceptible varieties under phytoplasma stress

To confirm the identities of these *ZjWRKY* genes for phytoplasma tolerance, we analysed the transcript profiles of *ZjWRKY* genes in a JWB-resistant variety and a susceptible variety (Fig. 7). The detection of the phytoplasma in the two varieties was shown in Additional file 4. After phytoplasma infection, the expression of 9 *ZjWRKYs* (*ZjWRKY2*, *9*, *22*, *24*, *29*, *34*, *36*, *42*, and *45*) in the susceptible variety were higher than in the resistant variety, and they were also upregulated in above diseased tissues (Fig. 6). In contrast, the expression of *ZjWRKY5* and *ZjWRKY49* in the resistant variety was higher than in the susceptible one during the early stages of infection. These two genes were significantly downregulated in diseased tissues. *ZjWRKY4* expression in the resistant variety was also higher than in the susceptible one. The above results indicated that some ZjWRKYs might play a role in phytoplasma tolerance.

## Discussion

In this study, a total of 54 WRKY-encoding genes were identified in the jujube genome. These genes can be can be divided into three groups (Group I to III), but this number may increase in the future once problems with the assembly and annotation of the jujube genome are addressed. As in other plants, almost all of the *ZjWRKY* genes share the WRKYGQK signature motif. However, the WRKYGKK variant was found in two jujube genes (Table 1, Fig. 2). Such slight variations in this region have also been reported in other plants such as Arabidopsis and apple [29].

Gene duplication events are the biggest contributors to the rapid expansion and evolution of gene families. Previous research has demonstrated that the Arabidopsis Group III WRKY gene family expanded rapidly as a result of recent segmental and tandem duplication events [30], and we found that this was also the case in the jujube genome. There are 6 tandemly duplicated *ZjWRKY* genes (*ZjWRKY47*, *ZjWRKY49*, *ZjWRKY50*, *ZjWRKY52*, *ZjWRKY53*, and *ZjWRKY54*) in Group III. The phylogenetic analysis (Additional file 5) indicated that 6 Group III *ZjWRKYs* were grouped and then clustered with 6 other genes from Arabidopsis; this also occurred in other subgroups from apple and pear. This finding suggests that the duplications in Group III *WRKY* genes occurred after the divergence of these plant species and tandem duplication events are the main contributors to the expansion of the Group III genes.

Previous research has demonstrated that Group I *WRKY* genes are the ancestors of the other *WRKY* genes in plants and are more likely to be constitutively expressed in different tissues [30]. In our study, the Group I genes and many genes from the other two groups were expressed in various tissues (Fig. 5), indicative of their diverse functions. These results provide some useful clues for additional investigations into the biological functions of these *WRKY* genes in jujube growth and development.

**Fig. 8:**
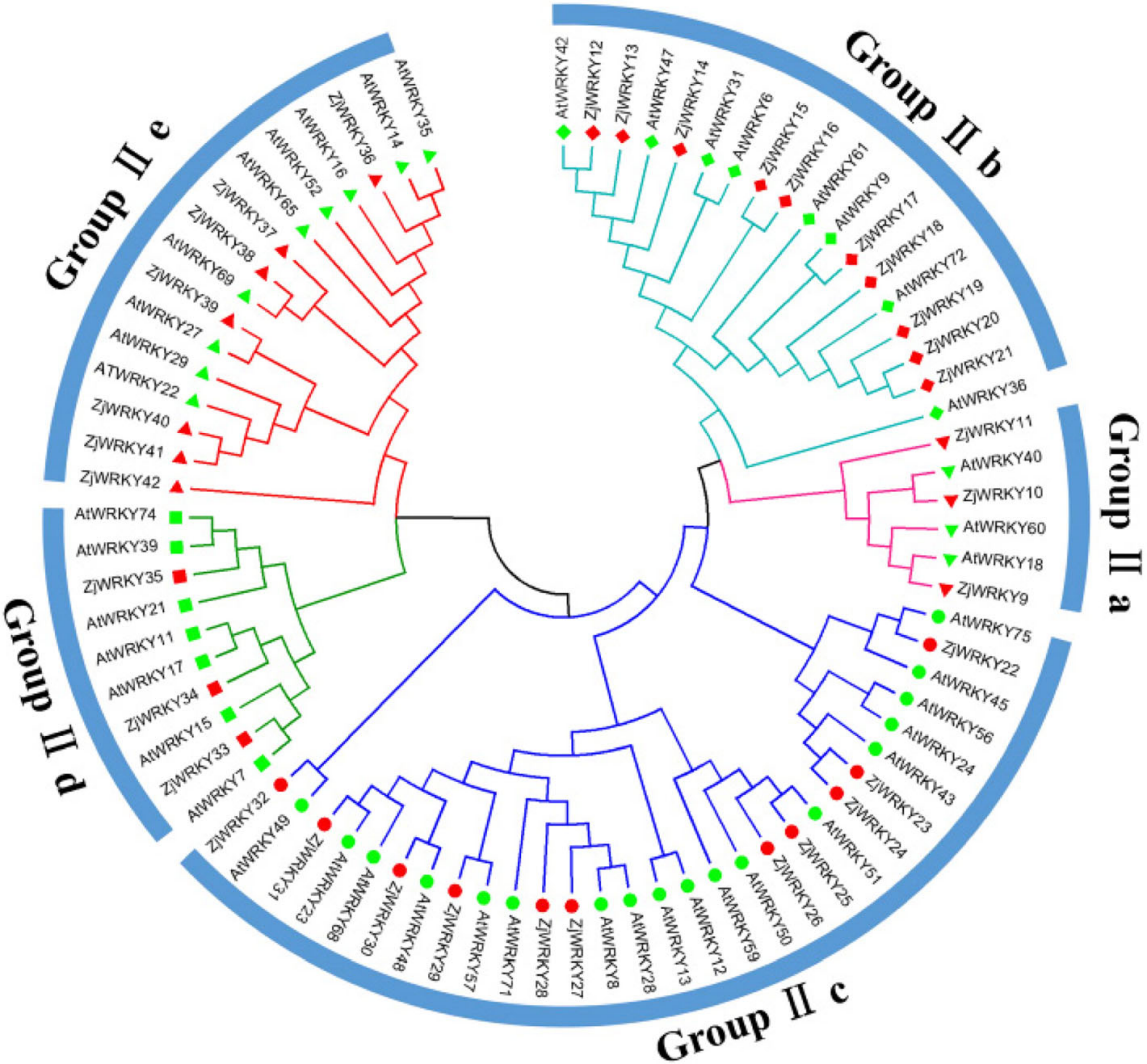
Phylogenetic relationships of GroupII *WRKY* genes from Chinese jujube and Arabidopsis

**Fig. 9:**
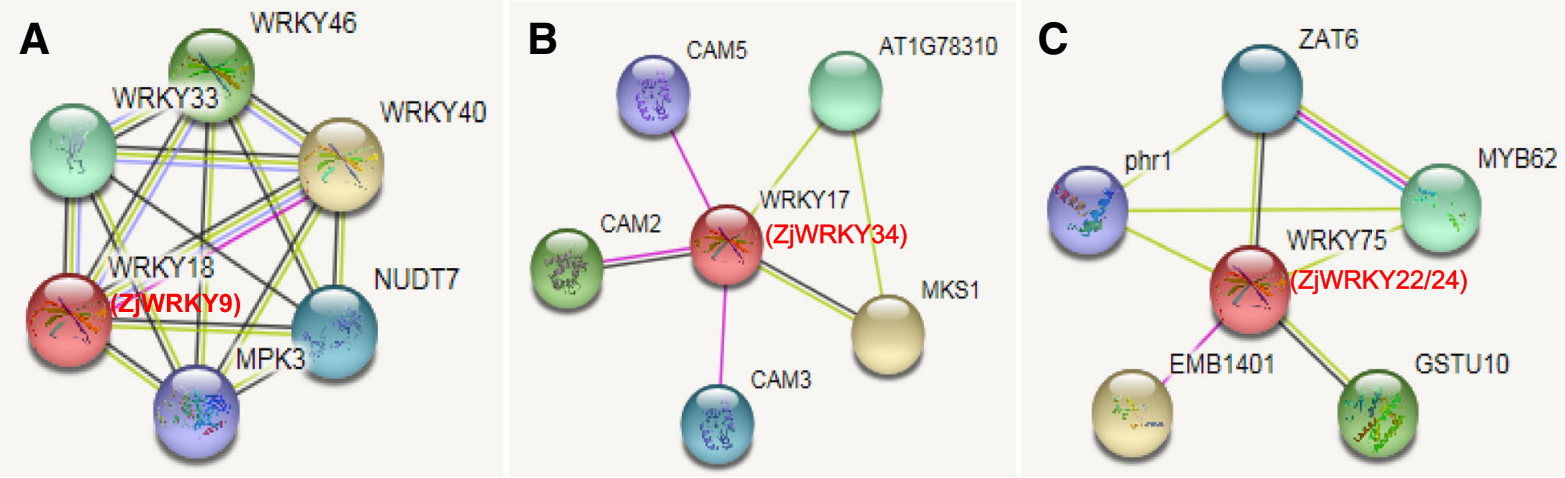
The protein-protein interaction analysis of ZjWRKY9, ZjWRKY34, ZjWRKY22 and ZjWRKY24 by STRING database. **a** ZjWRKY9 is the orthologous of AtWRKY18; **b** ZjWRKY34 is the orthologous of AtWRKY17; **c** ZjWRKY22 and ZjWRKY24 were the orthologous of AtWRKY75

Transgenic apple lines overexpressing *MdWRKY9* were significantly shorter and had significantly lower internode lengths than control plants [31], and its two orthologues in Arabidopsis (*AtWRKY11* and *AtWRKY17*) are negative regulators of basal resistance to a bacterial pathogen [32–34]. The Group II phylogenetic tree (Fig. 8) indicates that *AtWRKY11*, *17*, and *ZjWRKY34* are closely related. In this study, we found that *ZjWRKY34* was expressed at a noticeably higher level in infected jujube (Fig. 6) and in the JWB-resistant variety than in the susceptible variety at later stages of infection. STRING analysis showed that ZjWRKY34 (the orthologous of AtWRKY17) can interact with MSK1 and calmodulin (CAM, Fig. 9b). MKS1 is a regulator of plant defense response and it may contribute to MPK4-regulated defense activation by coupling the kinase to specific WRKY transcription factors. It also indicated that ZjWRKY34 might interact with the calmodulin-Ca^2+^ complex. Inferring the potential functions of *ZjWRKY34* from the known *AtWRKYs* suggests that *ZjWRKY34* might also act as a negative regulator in the defence process during jujube-phytoplasma interactions.

*ZjWRKY9* was actively expressed in JWB-diseased tissues. The phylogenetic tree (Fig. 8) and sequence alignment showed that *ZjWRKY9* was the orthologous gene of *AtWRKY18*. Transgenic *AtWRKY18* plants had increased expression of pathogenesis-related genes and resistance to the bacterial pathogen *Pseudomonas syringae*, indicating that *AtWRKY18* can positively modulate defence-related gene expression and disease resistance [12]. *AtWRKY18/40* act in a feedback repression system controlling basal defences [10]. In the other side, high *AtWRKY18* expression can cause severely abnormal plant growth [12]. These results suggest that proper expression of *ZjWRKY9* is critical for enhancing jujube’s defence response without negatively impacting plant growth. *ZjWRKY9*’s higher expression might be related to the abnormal growth of diseased jujube trees, such as witches’ broom and tiny leaves.

Overexpression of *AtWRKY28* and *AtWRKY75* induced an oxidative burst in host plants, which suppressed the hyphal growth of *Sclerotinia sclerotiorum* and consequently inhibited fungal infection [35]. STRING analysis predicted that AtWRKY75 could interact with GSTU10 (Fig. 9), which can eliminate the toxicity of oxygen bursts on plant cells and increase plant tolerance [36]. In this study, *ZjWRKY22* and *ZjWRKY24* are orthologous of *AtWRKY75* (Fig. 8), which behaved higher expression in the susceptible variety than in the resistant one (Fig. 7). That means that the two genes might eliminate the toxicity of oxygen bursts caused by phytoplasma infection. Previous study also indicated that the expression of *WRKY* gene was responsive to phytoplasma infection [37]. A divergent behaviour was previously observed for *OsWRKY28*. In rice, overexpression of *OsWRKY28* enhanced susceptibility to the rice blast fungus *Magnaporthe oryzae* and decreased accumulation of PR5 [38]. The knock-out of *OsWRKY28* led to a two-fold increase in resistance to a compatible rice blast fungus and this phenotype is accompanied by the increased expression of several defence-related genes [39]. Hence, *OsWRKY28* acts as a negative regulator of basal defence responses. Similarly, some *WRKY* genes might act as negative regulators of the basal resistance of jujube under phytoplasma stress, but further study is necessary to verify their specific functions.

## Conclusions

This paper described the WRKY gene family of Chinese jujube at the genome level. Their gene structure, chromosomal distribution, phylogenetic relationship, and tissue-specific expression patterns were presented in this study. Most of the ZjWRKYs were positive responses to phytoplasma invasion, and that provided meaningful candidates for the future studies of *ZjWRKYs* involved in jujube-phytoplasma interaction.

## Methods

### Plant material

The seven tissues including roots, young branches, old branches, leaves, flower buds, flowers and young fruits were collected from three jujube trees and used for organ-specific expression analysis.

Four kinds of tissues representing different degrees of JWB disease (apparently normal leaves (ANL), phyllody leaves (PL), and witches’-broom leaves (WBL)) from diseased trees, and healthy leaves (HL) from healthy trees were collected at four growth periods (June, July, August, and September). The All treatments were conducted with three biological replicates.

Phytoplasma cannot be cultured in vitro, and thus, JWB phytoplasma infection was transmitted by grafting. A JWB-resistant variety and a susceptible variety were used as scions for grafting onto JWB-diseased and healthy trees. All grafting treatments were conducted with three replicates. The samples were collected from sprouted scions at five growth periods (June, July, August, September and October). The samples were stored at − 80 °C until RNA extraction and expression analysis.

The JWB phytoplasma presence of the samples was detected by quantitative real-time PCR (qRT-PCR) [40]. The expression of phytoplasma *TMK* gene in jujube samples was analysed and *ZjACT* was used as an internal control.

### Identification and protein structure analysis of ZjWRKYs in Chinese jujube

First, *WRKY* genes from Arabidopsis were used as queries to search the jujube genome database. Next, the Pfam (http://pfam.xfam.org/) and SMART (http://smart.embl-heidelberg.de/) databases were used to confirm the predicted jujube WRKY proteins. To further confirm that the amino acid sequences in our data set were WRKYs, we manually examined the conserved WRKYGQK amino acid motif at the N-terminus and the zinc-finger-like motif at the C-terminus of the predicted WRKY domain. Truncated and false genes were excluded from our analysis. The number of amino acids, molecular weight, and theoretical pI of *ZjWRKY* genes were predicted by Protparam (https://web.expasy.org/compute_pi/). The conserved motifs of ZjWRKY proteins were detected by MEME (http://meme-suite.org/), using the following parameters: number of repetitions, any; maximum number of motifs, 20; and the optimum motif widths, 6–60 amino acid resides [41].

### The chromosomal location and gene structure of *ZjWRKYs*

To determine the chromosomal location of the *ZjWRKY* genes, their gene sequences were used as query sequences in BLASTN searches against the jujube genome. Each *ZjWRKY* gene was mapped to the jujube genome according to their genome coordinates. Tandem duplications were identified as previously described [42].

The website GSDS (http://gsds.cbi.pku.edu.cn/) was used to predict the number of exons from the coding domain sequences (CDS) and DNA sequences of the *WRKY* genes [43].

### Multiple sequence alignment and phylogenetic tree construction

The jujube WRKY proteins were classified into different groups based on their conserved domains. A phylogenetic tree was constructed from the amino acid sequences of WRKY conserved domains from jujube (54 sequences). The *Arabidopsis thaliana* WRKY proteins were retrieved from the TAIR database (http://www.arabidopsis.org/) as reported previously. Additionally, WRKY proteins of three other species (*Persica prunus* [22], *Pyres bretschneideri* [21], and *Malus domestica* [29]) were downloaded from NCBI. The classification of jujube Group II *WRKY* genes using the phylogenetic tree was dependent on the putative *Arabidopsis thaliana* orthologs. The MEGA 5.2 software and the neighbour-joining statistical method were used to construct a rooted phylogenetic tree [44–46]. The evolutionary distances were obtained using the p-distances method, and these distances were used to estimate the number of amino acid substitutions per site. The reliability of each phylogenetic tree was established by conducting 1000 bootstrap sampling iterations.

### RNA isolation and expression and statistical analysis

Total RNA was extracted using an RNAprep Pure Plant Kit (TIANGEN) according to the manufacturer’s protocol. After genomic DNA was removed by RNase-free DNase I (TIANGEN), RNA concentration and purity were checked on a NanoDrop2000 spectrophotometer. First-strand cDNA was synthesized by reverse transcribing 500 ng of total RNA with FastQuant RT Super Mix Kit (TIANGEN). The cDNA was used as the template for qRT-PCR.

Gene expression was detected by qRT-PCR. The primers used in this study are listed in Additional file 6. PCR products were amplified in triplicate using the Bio-Rad iQ™5 with TransStart Top Green qPCR SuperMix AQ131 (TransGen Biotech, China) in 20 μL reactions. Each reaction contained 10 μL of 2 × TransStart® Top Green qPCR SuperMix, 0.4 μL each of 10 μM primers, 8.2 μL of ddH_2_O and 1 μL of cDNA. The thermal profile for RT-qPCR was as follows: preincubation for 30 s at 95 °C, followed by 40 cycles of 5 s at 95 °C, 10 s at 53–58 °C, and 10 s at 72 °C. Three biological replicates were performed for each treatment. Threshold cycle values were calculated using iCycler software, and *ZjACT* was used as an internal control [47]. Relative transcript levels were calculated according to the 2^–ΔΔCT^ method [48].

### Yeast two-hybrid screening (Y2H)

ZjWRKY protein is fused to the Gal4 DNA-binding domain (BD) and the screening proteins are fused to the Gal4 activation domain (AD). The AD-fused ZjWRKY and BD-fused ZjMAPK were amplified using the primers shown in Supplementary Table S1, and cloned into the pGADT7 vector and pGBKT7 respectively. ZjWRKYs were digested by SmaI and the ZjMAPKs were digested by EcoRI and co-transformed AH109 stain with pairs of appropriate pGADT7 and pGBKT7 vectors. Successful co-transformants were selected on synthetically defined medium lacking tryptophan and leucine (SD/−Trp/−Leu). To examine protein-protein interactions, freshly transformed yeast colonies were resuspended in 10 μL sterile deionized water, and 0.5 μL aliquots were spotted upon medium lacking leucine and tryptophan (−LW) and medium lacking leucine, tryptophan, histidine (−LWH), supplemented with 7 mM 3-Amino-1,2,4-triazole (3-AT; Sigma Aldrich) (−LWH + 3AT) and medium lacking leucine, tryptophan, histidine, adenine (−LWAH). Growth was scored after 3 d of incubation at 28 °C.

## Additional files

Additional file 1: Number of WRKY gene family from Chinese jujube and other species (DOC 28 kb)

Additional file 2: The amino acid sequences of 8 motifs among ZjWRKY proteins. (DOC 160 kb)

Additional file 3: Positions of 13 *ZjWRKY* genes on the jujube scaffolds. The jujube scaffolds were arranged in a circle. (DOC 319 kb)

Additional file 4: Expression analysis of phytoplasma TMK in four kinds of leaves (A) and in susceptible and resistant varieties (B). (DOC 31 kb)

Additional file 5: The phylogenetic analysis of Group III WRKYs of *Ziziphus jujuba*, *Arabidopsis thaliana*, *Pyres Bretschneideri*, *Persica Prunus* and *Malus domestica*. (DOC 89 kb)

Additional file 6: The primers of *ZjWRKY* genes used in this study. (DOC 36 kb)

## Abbreviations

ANL: Apparently normal leaves
CDS: Coding domain sequences
HL: Healthy leaves
JWB: Jujube witches’ broom disease
PL: Phyllody leaves
qRT-PCR: Quantitative real-time PCR
TFs: Transcription factors
WBL: Witches’-broom leaves
