# Supplementary material for: Genome-wide analysis of the WRKY gene family and their positive responses to phytoplasma invasion in Chinese jujube
Source: BMC Genomics. 2019 Jun 7;20:464. doi: 10.1186/s12864-019-5789-8 (PMC6555936; doi:10.1186/s12864-019-5789-8)
Supplement: Supplementary file 1 — Number of WRKY gene family from Chinese jujube and other species (DOC 28 kb) [file 12864_2019_5789_MOESM1_ESM.doc]

| **Group** | **Subgroup** | **Gene number** | | | | | |
| --- | --- | --- | --- | --- | --- | --- | --- |
|  |  | **AtWRKY** | **PpWRKY** | **MdWRKY** | **VvWRKY** | **ZjWRKY** | **OsWRKY** |
| **Ι** |  | **14** | **10** | **22** | **13** | **8** | **15** |
| **Ⅱ** | **Ⅱa** | **3** | **3** | **9** | **3** | **3** | **4** |
|  | **Ⅱb** | **8** | **8** | **28** | **8** | **10** | **8** |
|  | **Ⅱc** | **18** | **15** | **13** | **16** | **11** | **15** |
|  | **Ⅱd** | **7** | **7** | **13** | **6** | **3** | **7** |
|  | **Ⅱe** | **9** | **7** | **16** | **6** | **7** | **11** |
| **Ⅲ** |  | **13** | **8** | **14** | **6** | **12** | **36** |
| **Ⅳ** |  |  |  | **13** | **2** |  |  |
| **Total** |  | **72** | **58** | **127** | **59** | **54** | **96** |
