# Supplementary figures and images for: Genome-wide analysis of the WRKY gene family and their positive responses to phytoplasma invasion in Chinese jujube
Source: BMC Genomics. 2019 Jun 7;20:464. doi: 10.1186/s12864-019-5789-8 (PMC6555936; doi:10.1186/s12864-019-5789-8)

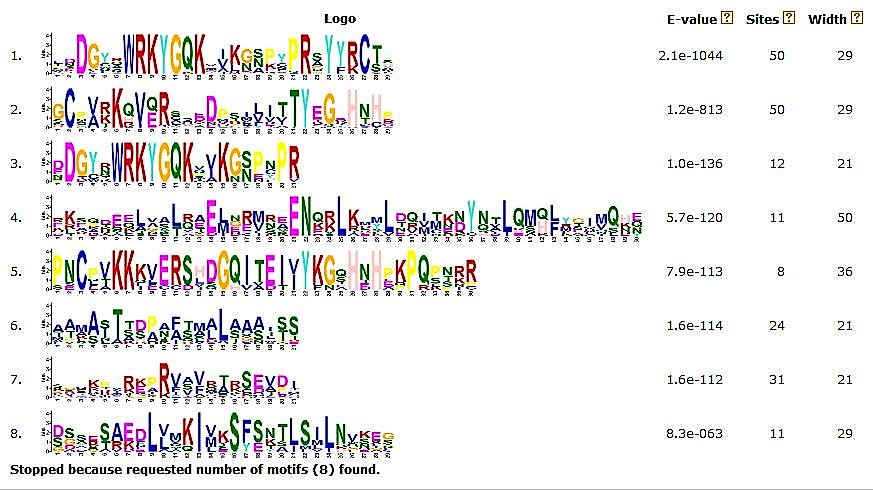

Supplement: Supplementary file 2 — The amino acid sequences of 8 motifs among ZjWRKY proteins. (DOC 160 kb) [file 12864_2019_5789_MOESM2_ESM.doc]

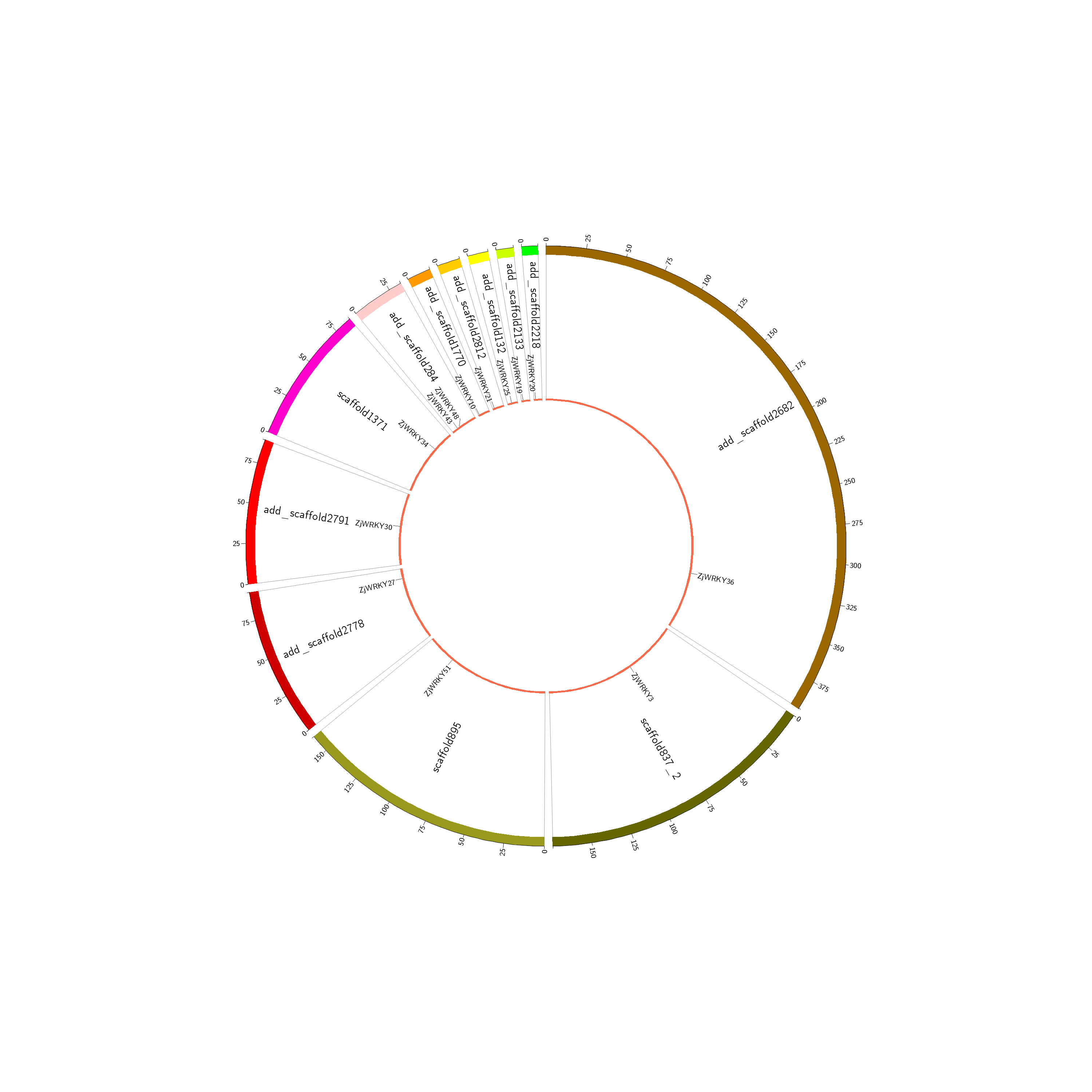

Supplement: Supplementary file 3 — Positions of 13 ZjWRKY genes on the jujube scaffolds. The jujube scaffolds were arranged in a circle. (DOC 319 kb) [file 12864_2019_5789_MOESM3_ESM.doc]
