# Supplementary material for: Genome-wide analysis of the WRKY gene family and their positive responses to phytoplasma invasion in Chinese jujube
Source: BMC Genomics. 2019 Jun 7;20:464. doi: 10.1186/s12864-019-5789-8 (PMC6555936; doi:10.1186/s12864-019-5789-8)
Supplement: Supplementary file 5 — The phylogenetic analysis of Group III WRKYs of Ziziphus jujuba, Arabidopsis thaliana, Pyres Bretschneideri, Persica Prunus and Malus domestica. (DOC 89 kb) [file 12864_2019_5789_MOESM5_ESM.doc]

*Ziziphus jujuba*

*Arabidopsis thaliana*

*Pyres Bretschneideri*

*Persica Prunus*

*Malus Domestica*
