# Supplementary material for: Genome-wide analysis of the WRKY gene family and their positive responses to phytoplasma invasion in Chinese jujube
Source: BMC Genomics. 2019 Jun 7;20:464. doi: 10.1186/s12864-019-5789-8 (PMC6555936; doi:10.1186/s12864-019-5789-8)
Supplement: Supplementary file 6 — The primers of ZjWRKY genes used in this study. (DOC 36 kb) [file 12864_2019_5789_MOESM6_ESM.doc]

| Gene | 5’Primer | 3’Primer |
| --- | --- | --- |
| *ZjWRKY-1* | AACTCCCGATAACTCTTGTG | GGTTCTTGAATACCCTCCC |
| *ZjWRKY-2* | AGGGTATTCAGCTTCTGGGA | CGCTCCACATGTTTCCTCAC |
| *ZjWRKY-3* | GTGGGAATAACTTGTGCCG | GATGAGACAGGGTTTGCTTG |
| *ZjWRKY-4* | TGATCCCGACTCGAAACGAC | TCCTTGGGTTTGGATTGCCT |
| *ZjWRKY-5* | TGATGGATACAGATGGCGCA | CACGGCTGTTTCAATGTGCT |
| *ZjWRKY-6* | TAGCCATAACCATCCCAAGCC | GTGTAGCAGCAGAATCCGTC |
| *ZjWRKY-7* | TCCTATGACCCTTGTTTCC | GTTGCTACACCTCCTTCCT |
| *ZjWRKY-8* | TTCTGAGGGAAGGACCGATG | AAAGCTCTGGAGTTTCGGCT |
| *ZjWRKY-9* | CGGACTGACTCATCCAATA | CCTTCATAAGTAGCCACCAA |
| *ZjWRKY-10* | TCGTTGCATGACCACAACAC | GAAATGTCGAACTTCCGGCG |
| *ZjWRKY-13* | CTCCATTCCCAACGGTGACA | GCAATGAAGGCGTGGATGAC |
| *ZjWRKY-15* | ACCCTCGACCTCACCCATAA | AGTACATGGGGTGCCCTAGA |
| *ZjWRKY-18* | ACAGGGATGAGCTTGAAGTGG | TGGACGATTTGTTCAAGCAGC |
| *ZjWRKY-22* | ATAGGTGCACGCATCAAGGT | GCATCTGGCTCAAGATATGCTC |
| *ZjWRKY-24* | AGACGAAATCGTCATCGGGAA | TGCATCCTCGATGAGTACACC |
| *ZjWRKY-26* | GAAGAACAGCCCAAATCC | CCCTCGTAGGTGGTAATC |
| *ZjWRKY-29* | GAATTTGGGAGGGACGTTGC | TCGCTAGAGCTCGAAGACAC |
| *ZjWRKY-32* | CCACCTTCACTTTGCGTACC | CACTACCTGGGCCCATTTCT |
| *ZjWRKY-33* | AGTACGGCCAGAAACCCATC | TGATTGTGGTCGCCTTCGTA |
| *ZjWRKY-34* | GAGCTCGTCGTTCATGTCCT | CAGACCCGGATCCAGAGAAC |
| *ZjWRKY-35* | ATGCGGTAGCAGTGGTAGA | CACGAATAATCATCAGGAGG |
| *ZjWRKY-36* | ACCAAACAGCAGACCGAGTG | GTTTTCTTGCCGAACAGCCC |
| *ZjWRKY-37* | GTCCACGGTTCTTGAAAGCC | AGTGATTCGTCCTCTTCCCG |
| *ZjWRKY-38* | GGAATACCCTGAACCCG | CCTCCAAGCCCACGAAT |
| *ZjWRKY-41* | GTGACGATTTCTTTGTGGG | CGTGGCAGCATTATTAGC |
| *ZjWRKY-42* | TGCAGTACATCGAAGGGGTG | ATCAGCGAGATTAGCGGTGG |
| *ZjWRKY-44* | AGAGAGGTTGTTGCACCTCC | CAAGGTTGTTCATCCGGCTC |
| *ZjWRKY-45* | GGTGGACACAGCAAGTGAGA | GTTGGATCCTTGTCGGAACG |
| *ZjWRKY-47* | TGTCAAAGATCGGAGGGGTTG | GTTTGGTTGCTTGGCACTGT |
| *ZjWRKY-48* | AGATGGCGACTCAGCTTCAG | GGCATGAGAGGACTCGGTTA |
| *ZjWRKY-49* | TTAGCCTCTCGAACCCAAGC | GACCCCTGCGGTCTTTAACA |
