# Supplementary material for: Genome-wide analysis of the WRKY gene family and their positive responses to phytoplasma invasion in Chinese jujube
Source: BMC Genomics. 2019 Jun 7;20:464. doi: 10.1186/s12864-019-5789-8 (PMC6555936; doi:10.1186/s12864-019-5789-8)
Supplement: Supplementary file 5 [file 12864_2019_5789_MOESM4_ESM.doc]

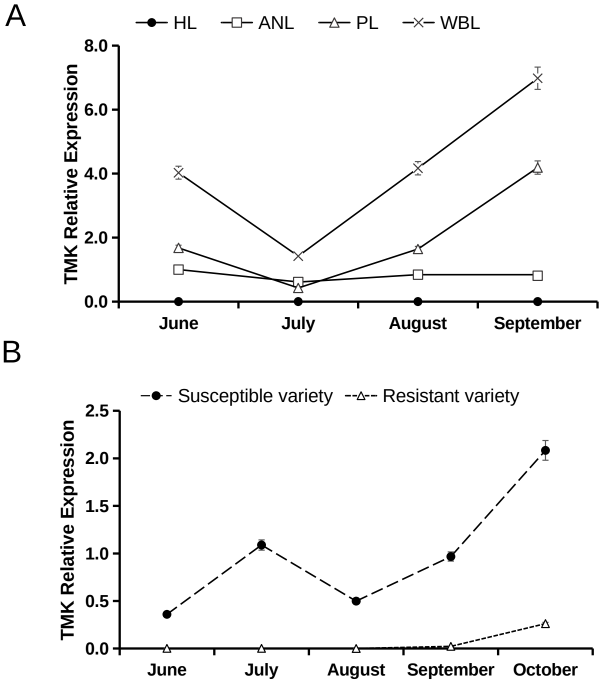

A
HL
ANL
PL
WBL
TMK Relative Expression
8.0
6.0
4.0
2.0
0.0
June
July
August
September
B
Susceptible variety
Resistant variety
TMK Relative Expression
2.5
2.0
1.5
1.0
0.5
0.0
June
July
August
September
October
